# Comparison of EuroSCORE II and STS Risk Scoring Systems in Patients who Underwent Open-heart Surgery

**DOI:** 10.4274/TJAR.2025.241778

**Published:** 2025-07-24

**Authors:** Erkam Saka, Esin Öztürk, Aslıhan Esra Yüksel, Nüzhet Seden Kocabaş

**Affiliations:** 1 Ege University Faculty of Medicine Department of Anaesthesiology and Reanimation, İzmir, Türkiye

**Keywords:** EuroSCORE II, open-heart surgery, preoperative evaluation

## Abstract

**Objective:**

In the present study, European Cardiac Operative Risk Assessment System II (EuroSCORE II) and the Society of Thoracic Surgery (STS) risk scoring systems were used to predict mortality in patients who underwent various types of open-heart surgery, including coronary artery bypass grafting, aortic valve replacement, mitral valve replacement, and combined valve surgery with coronary artery bypass grafting, in the cardiovascular surgery operating room. The aim was to compare risk assessment systems regarding their clinical applicability.

**Methods:**

A total of 469 patients, 141 (30.1%) female and 328 (69.9%) male, were included in the study. All risk factors were retrospectively recorded according to the EuroSCORE II and STS risk assessment systems. Statistical analysis was performed using the receiver operating characteristic (ROC) curve. Predicted and actual mortality rates were compared for each risk-scoring system.

**Results:**

When the ability of the EuroSCORE and STS risk classifications to predict mortality was analyzed using the ROC curve, the area under the curve for the EuroSCORE II risk score was 78.3% (*P* < 0.001), while the area under the curve for the STS risk score was 82.3% (*P* < 0.001). In our study, the STS scoring system was found to have a greater predictive value than EuroSCORE II. When the patients’ observed and expected mortality rates were examined according to the EuroSCORE II and STS risk scores, no statistically significant relationship was found between the expected and observed mortality rates for each risk group.

**Conclusion:**

In our study, the STS risk scoring system was found to be more accurate in predicting in-hospital mortality than the EuroSCORE. However, there was no statistically significant difference between the expected and observed mortality rates in either risk-scoring system. There is no consensus in the literature regarding which scoring system is more effective. More studies from different societies are needed.

**Main Points**

• We have found that the European Cardiac Operative Risk Assessment System II (EuroSCORE II) risk classification system’s predictive ability was lower (70-80%) than that of the the Society of Thoracic Surgery (STS) system (80-90%).

• The observed mortality rate was found to be high for both the EuroSCORE II and STS risk scoring systems when compared to the expected mortality rates.

## Introduction

Despite advanced surgical and anaesthetic techniques, open-heart surgery has a mortality rate of up to 4% and carries the risk of both cardiac and non-cardiac complications. Studies have shown that cardiac complications (62.1%) are the leading cause of mortality. Other complications associated with mortality include respiratory complications (11.8%), infections (7.7%), and acute neurological injury (6%).^1^

Although many risk scoring systems have been developed to determine the risk of mortality and morbidity in open-heart surgery, the most widely used are the European Cardiac Operative Risk Assessment System II (EuroSCORE II) and the Society of Thoracic Surgeons (STS) scoring systems.^2^

The original version of EuroSCORE was drawn from a European database of more than 19,000 cardiac surgery patients, most of whom had undergone cardiac surgery between 1995 and 1999. Approximately one-third of the patients included in this database had undergone coronary artery and valve surgeries.^3, 4, 5^ EuroSCORE has gained wide acceptance since its publication and has been used extensively in the care of cardiac surgery patients, both to assess risk and to publicize improved operations.^6^ However, over time, the necessity of making improvements in practice has emerged.^6, 7^ New variables, including creatinine clearance and liver function, were added to the EuroSCORE II risk analysis, and the relative weighting or impact of these variables was adjusted simultaneously.^4^

EuroSCORE II evaluates risk factors related to the patient, their cardiovascular status, and the surgery performed, and it produces a risk score. When the data are evaluated, risk scores are classified as low (score: 0-2), moderate (score: 3-5), and high (score >6).

The STS risk model was created using data from its own database. The most recent update was created in 2018 by studying 579,335 cases between 2011-2014 and 670,830 cases between 2014-2016.^8, 9^ Patients were assessed for 65 risk factors. This scoring system provides a risk score associated with various complications: operative mortality (all deaths occurring within 30 days of surgery), stroke (acute onset focal or global neurological dysfunction occurring within 24 hours), renal risks including risk, injury, failure, loss, end-stage renal disease, prolonged ventilation or reintubation (>24 hours), mediastinitis or deep sternal wound infection, reoperation due to bleeding or tamponade, major morbidity and mortality, prolonged postoperative hospital stay (PLOS) >14 days, and short PLOS <6 days.^9^

Both scoring systems were developed in developed countries such as Europe and the United States, and there are questions about whether they reflect the true mortality and morbidity risk for each society. In addition, there is an ongoing debate about which scoring system provides more accurate statistical results and is therefore more effective.^10, 11, 12^ In our study, we aimed to determine which of the EuroSCORE II and STS risk scoring systems is superior in predicting the risk of postoperative mortality and morbidity in patients undergoing open-heart surgery by comparing them using a retrospective chart review method. We also aimed to determine their suitability for Turkish society.

## Methods

All patients older than 18 years who underwent open-heart surgery [coronary artery bypass grafting (CABG), aortic valve replacement, mitral valve replacement or repair, or combined valve surgery with CABG] in the cardiovascular surgery operating room of Ege University Faculty of Medicine Hospital between 2019 and 2020 were included in our study. Our study is cross-sectional, and no sample selection method was used. After approval from the Ethics Committee of Ege University Faculty of Medicine Hospital (approval no.: 20-8T/17, date: 05.08.2020), the preoperative, intraoperative, and postoperative data of the patients who gave informed consent were retrospectively retrieved from the medical records.

Patients under 18 years of age, with missing data on surgery, requiring renal dialysis due to preoperative renal failure, undergoing off-pump heart surgery, intubated before surgery, undergoing emergency surgery, or undergoing surgery with inotropic support were excluded from the study.

In addition to patient demographics, preoperative risks such as unstable angina, previous myocardial infarction, acute myocardial infarction (<3 weeks), low left ventricular ejection fraction (<35%), diabetes mellitus, hypercholesterolemia, hypertension, peripheral vascular disease, cerebrovascular disease, respiratory disease, alcohol consumption, and smoking habits were recorded. Factor-determining data were recorded. Patients’ EuroSCORE II and STS Risk Scoring System scores were calculated and recorded. Postoperative cardiac complications (myocardial infarction, atrial and ventricular arrhythmias, need for more than two inotropic agents or mechanical circulatory support, respiratory complications, cerebrovascular complications, postoperative renal dysfunction, gastrointestinal complications, sepsis, multiple organ failure, sternal infection, need for reoperation) were recorded. In addition, postoperative data such as mechanical ventilation, intensive care, hospital stay, and in-hospital mortality were retrospectively collected.

### Statistical Analysis

Patient data collected in the study was analyzed using IBM Statistical Package for the Social Sciences (SPSS 23.0-IBM, NY, USA). Categorical frequencies and percentages for categorical data, as well as means and standard deviations for continuous data, are provided. Receiver operating characteristic (ROC) was used to determine the predictive value of risk scores for mortality. The analysis was performed. Effect of each patient’s risk score on mortality logistic regression. It was calculated using analysis. For comparison between groups, the “Independent Samples t-test”, "chi-squared test”, “chi-square”, or “Fisher’s exact test” were used for comparisons between groups. Results are considered statistically significant if the *P *value is less than 0.05.

## Results

The study included 469 cases, 141 female (30.1%) and 328 (69.9%) male patients. Table 1 shows the distribution of the patient’s demographic and clinical findings. The distribution of preoperative comorbidities of the patients is given in Table 2. The diagnosis distribution of the patients included in the study and the mortality rates according to diagnosis are shown in Table 3. The highest mortality rate was for CABG (n = 338; 72.1%) (Table 4).

**Figure 1:**
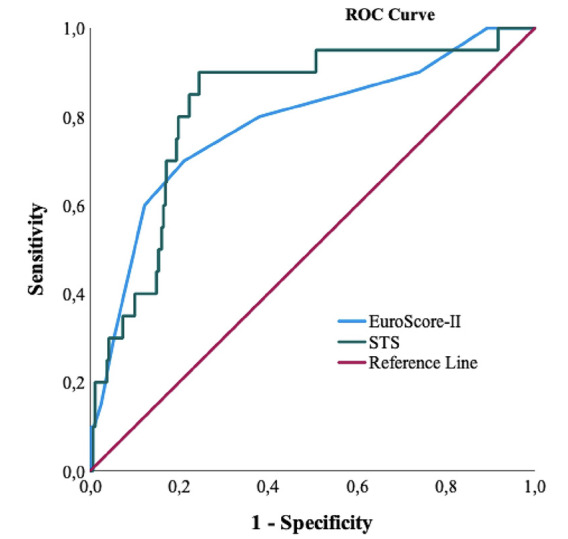
ROC curve sensitivity and 1 - specifity. ROC, receiver operating characteristic; STS, Society of Thoracic Surgeons Score; EuroSCORE II, European Cardiac Operative Risk Assessment System II.

When the risk scores of the patients were examined, the mean EuroSCORE II, and STS scores were 3.1±2.2 and 7.5±5.3, respectively (Table 5). According to the EuroSCORE II and STS scoring systems, 14.3% (n = 67); and 30.3% (n = 142) of the patients were in the high-risk category, respectively. When examined using the ROC curve analysis method (Figure 1), the area under the curve (AUC) for the EuroSCORE II risk scores was 78.3% with a cut-off value of 4. The AUC for the STS risk score was found to be 82.3% with a cut-off value of 8.45 (Table 6). In other words, the STS score showed a significantly higher accuracy in predicting mortality risk compared to the EuroSCORE II, in our study (AUC: 0.823-0.783, *P *< 0.05).

When the impact of EuroSCORE II and STS risk scores on predicting mortality was examined, it was determined that a one-unit increase in EuroSCORE II risk scores increased the mortality risk (odds ratio) by 1.5 [confidence interval (CI): 1.3-1.8] times (*P *< 0.05), and one-unit increase in STS risk scores increased the mortality risk (odds ratio): by 1.12 (CI: 1.10-1.18) times (*P *< 0.05).

Observed and expected mortality rates according to the EuroSCORE II and STS risk scores of the patients are given in Table 7. A statistically significant relationship does not exist between the expected and observed mortality rates for each risk group (*P* > 0.05).

## Discussion

The use of risk assessment methods before cardiovascular surgery makes it possible to predict postoperative mortality and complications, to better inform patients and their families about possible problems after surgery, and to take the necessary precautions.

The EuroSCORE II and STS scoring systems are two of the most widely used validated risk assessment methods.^13, 14^ EuroSCORE was originally developed in Europe and is widely used throughout the world.^15^ The STS risk scoring system is more widely used in North America. Both risk scores are robust predictors of postoperative mortality. However, each society has a different socio-economic and cultural structure. Both scoring systems were developed in countries such as North America and Europe. There is ongoing debate about their suitability in developing societies and about which is more effective.

Mandel et al.,^16^ reported in 2003 that both the EuroSCORE and STS scoring systems needed improvement, but they were indispensable to surgeons at the time of the study. Considering 9,248 patients evaluated with EuroSCORE in 35 cardiac centers in the People’s Republic of China, the authors argued that this scoring system could not be used to predict outcomes after CABG surgery and that another scoring system was needed for their race.^17^

In a large-scale study conducted by Shales et al.^11^ in India in 2021, it was concluded that EuroSCORE was pessimistic in predicting mortality, while the STS risk score underestimated mortality. When the discrimination power was examined, it was determined that both risk-scoring systems had equal and sufficient power.

In another study published in 2024 on 438 patients in Brazil, Wolf and Amato^18^ determined the AUC value for the discrimination power of the STS risk scoring system as 0.646 and for EuroSCORE as 0.697, emphasizing that it did not provide optimum results. In a different study conducted in China by Gao et al.^12^, it was determined that both the EuroSCORE and STS risk scoring systems had sufficient discrimination power. In this study, EuroSCORE II was found to be superior to the STS risk scoring system in terms of predicting mortality.^12^

Studies conducted in different centers in Türkiye have shown that the EuroSCORE II risk classification is reliable in predicting the risk of mortality associated with cardiovascular surgery.^19^ Kandemir et al.^20^ found the AUC-ROC curve to be 0.83 for the EuroSCORE II risk assessment system and 0.82 for the STS risk assessment system in cases of patients undergoing isolated CABG.^20^ The sample size of the study by Kandemir et al.^20^ was limited to 148 patients, which is smaller than that of our study.

In studies evaluating the mortality risk of transcatheter aortic valve replacement, Sedaghat et al.^21^ demonstrated the predictive superiority of EuroSCORE II over the STS scoring system in a cohort of 206 patients, and similarly, Stähli et al.^22^ demonstrated this in a cohort of 350 patients. However, Hemmann et al.,^23^ reported superiority of STS in their study of 426 patients. A study comparing STS and EuroSCORE II scoring systems in Pakistan showed that EuroSCORE II was superior in isolated valve surgery cases, while STS provided better predictive data for coronary artery bypass and valve surgery.^24^

In our study, when we examined the EuroSCORE II and STS risk scoring systems using the ROC curve, the AUC value for the STS risk scoring system was 0.823 and the AUC value for the EuroSCORE was 0.783. While the EuroSCORE risk scoring system showed a moderate level of discrimination (70-80%), the STS system showed better discrimination (80-90%). The STS score was a better predictor than the EuroSCORE II.

When the observed and expected mortality rates of the patients in our study were examined according to the EuroSCORE II and STS risk scoring systems, the expected and observed mortality rates for the high-risk patient group were 14% and 17.9%, respectively, according to EuroSCORE II. In contrast, the respective percentages according to the STS scoring system were 8.1% and 12.7%. We attribute the different results of these two risk scoring systems, which were developed using large databases, to the fact that the countries where they were developed are ahead of Türkiye in terms of socioeconomic and medical facilities, although the differences were not statistically significant (*P *> 0.05). When Kandemir et al.^20^ compared the two scoring systems in 148 patients undergoing CABG surgery, they found no statistically significant difference between the expected and actual mortality rates for both systems. More comprehensive and multicentre studies are needed for the Turkish community and other developing societies.

The adoption of a single system to assess mortality risk worldwide will be of great convenience to physicians. However, scoring systems differ in the way they assess some risks. Each system has its own shortcomings and limitations. Given that access to health care and medical facilities is not homogeneous worldwide, along with the genetic and geographical differences of patients, more multicentre studies are needed to determine which risk scoring system is more useful and applicable in Turkish society. Furthermore, since EuroSCORE II was developed in 2012 and the STS risk scoring system in 2018, an updated evaluation is still needed regarding the applicability of these two risk scoring systems in light of the developing technology in cardiovascular surgery and anaesthesia. In conclusion, in our study, the STS score showed a better predictive performance than the EuroSCORE II.

Therefore, it is recommended to consider STS more in clinical use. Although there is no statistically significant difference between the observed and expected mortality rates, the STS risk scoring system and the EuroSCORE II scoring system still represent the best results in this field worldwide and should continue to be used until a more optimal risk scoring system is developed.

### Study Limitations

Our study was conducted retrospectively and as a single-center study. Due to the retrospective nature of our study, patients had to be excluded from the study because of insufficient patient file data. Since our study was single-center, it may not reflect the situation nationwide. Our study focused on in-hospital mortality and did not evaluate either risk scoring system regarding 5-year survival. Additional studies are needed on this subject.

## Conclusion

In our study, the STS risk scoring system showed a better predictive performance than EuroSCORE II. Therefore, it is recommended to consider the use of STS more frequently in clinical practice. In cardiac surgery, patients should be approached with a multidimensional strategy from the preoperative period. Additional precautions should be taken in the high-risk patient group using risk scores in collaboration with the surgical team.

## Ethics

**Ethics Committee Approval:** Ethical approval was obtained from the Ethics Committee of Ege University Faculty of Medicine Hospital (approval no.: 20-8T/17, date: 05.08.2020).

**Informed Consent:** The preoperative, intraoperative, and postoperative data of the patients who gave informed consent were retrospectively retrieved from the medical records.

## Figures and Tables

**Table 1. Demographic, and Clinical Characteristics of the Patients:** 

**Characteristic features**		-
Gender	Female, n (%)	141 (30.1)
	Male, n (%)	328 (69.9)
Age, years (mean ± SD)		63±11.2
Height, cm (mean ± SD)		170±9.1
Body weight, kg		76±11.6
Tobacco use, n (%) (mean ± SD)		267 (56.9)
Left ventricular EF%	>50, n (%)	299 (63.8)
	35-50, n (%)	145 (30.9)
	<35, n (%)	25 (5.3)
Duration of anaesthesia (mean ± SD)		364±58.4
Duration of surgery (mean ± SD)		332±57.4
Arotic cross-clamp time, min. (mean ± SD)		69±31.0
Duraion of CPB, min. (mean ± SD)		99±37
Duration of mechanical ventilation, hrs (mean ± SD)		0.47±2.7
ICU stay, days (mean ± SD)		2.1±3.8
Hospital stay, days (mean ± SD)		12.3±7.6

SD, standard deviation; EF, ejection fraction; CPB, cardiopulmonary bypass; ICU, intensive care unit

**Table 2. Distribution of Patients’ Preoperative Comorbidities:** 

**Preoperative comorbidities**	**n (%)**
CAD	366 (78.2)
Hypertension	310 (66.1)
DM	193 (41.2)
Unstable angina	166 (35.5)
Hyperlipidemia	144 (30.7)
COPD	52 (11.1)
Arrhytmia	35 (7.5)
Acute MI <3 weeks	34 (7.2)
History of SVO	26 (5.6)
EF% <35	25 (5.3)
PAH	15 (3.2)
Carotid artery disease	14 (3)
History of MI	9 (1.9)
CRF	3 (0.6)

CAD, coronary artery disease; DM, diabetes mellitus; COPD, chronic obstructive pulmonary disease; EF, ejection fraction; PAH, pulmonary arterial hypertension; MI, myocardial infarction; CRF, chronic renal failure

**Table 3. Distribution of Patients According to Their Diagnoses:** 

**Diagnoses**	**n (%)**	**Exitus n (%)**
CAD	339 (72.3)	14 (70.0)
MI	26 (5.5)	0 (0.0)
MS	15 (3.2)	0 (0.0)
AI	10 (2.1)	1 (5.0)
AS	22 (4.7)	1 (5.0)
MI+AI	21 (4.5)	0 (0.0)
MS+MI	2 (0.4)	0 (0.0)
CAD+AS	17 (3.6)	1 (5.0)
AS+AI	2 (0.4)	0 (0.0)
CAD+MI	12 (2.6)	2 (10.0)
MI+TI	2 (0.4)	1 (5.0)
CAD+AI	1 (0.2)	0 (0.0)
Total	469 (100.0)	20 (100.0)

CAD, coronary artery disease; MI, mitral insufficiency; AI, aortic insufficiency; AS, aortic stenosis; TI, tricuspid insufficiency; MS, mitral stenosis

**Table 4. Distribution of Patients by Operation Types:** 

Type of surgery*	**n (%)**	**Exitus n (%)**
CABG	338 (72.1)	14 (70.0)
MVR	46 (9.8)	0 (0.0)
AVR	34 (7.2)	2 (10.0)
AVR+CABG	17 (3.6)	1 (5.0)
MVR+CABG	13 (2.8)	2 (10.0)
MVR+AVR	19 (4.1)	0 (0.0)
MVR+TVR	2 (0.4)	1 (5.0)
Total	469 (100.0)	20 (100.0)

*CABG, coronary artery bypass grafting; MVR, mitral valve replacement; AVR, aortic valve replacement; TVR, tricuspid valve replacement

**Table 5. Distribution of Risk Scores of the Patients:** 

**Characteristic features**	
**EuroSCORE II (3.1±2.2)**	**n (%)**
Low	199 (42.4)
Intermediate	203 (43.3)
High	67 (14.3)
STS	7.5±5.3
Low	91 (19.4)
Intermediate	236 (50.3)
High	142 (30.3)
**The risk of re-operation according to STS**	
Low	447 (95.3)
Intermediate	22 (4.7)
Re-operation	30 (6.4)

STS**, **Society of Thoracic Surgeons Score; EuroSCORE II, European Cardiac Operative Risk Assessment System II

**Table 6. ROC-curve Estimates of Survival Rates According to EuroSCORE II and STS Scoring Systems:** 

**Risk scoring system**	**AUC (CI 95%)**	**Cut-off value**	***P *value**	**Sensitivity (%)**	**Specificity (%)**	**PPD(%)**	**NPD(%)**
EuroSCORE II	0.783 (0.742-0.819)	>4.000	<0.001	70.0	78.8	12.8	98.3
STS	0.823 (0.785-0.856)	>8.450	<0.001	90.0	75.5	14.1	99.4

ROC,receiver operating characteristic; STS, Society of Thoracic Surgeons Score; AUC, area under curve; CI, confidence interval; PPD, positive predictive value; NPD, negative predictive value; EuroSCORE II, European Cardiac Operative Risk Assessment System II

**Table 7. Evaluation of Observed and Expected Mortality Rates According to EuroSCORE II and STS Risk Scoring Systems:** 

**Risk scoring system**	**Risk group**	**Patients (n)**	**Observed mortality (n)**	**Observed mortality (%)**	**Expected mortality (%)**	***P *value**
EuroSCORE II	Low	199	3	1.5	1.3	1.000
	Intermediate	203	5	2.5	3.9	0.575
	High	67	12	17.9	14.2	0.635
STS	Low	91	1	1.1	2.1	1.000
	Intermediate	236	1	0.4	2.8	0.068
	High	142	18	12.7	8.1	0.170

STS, Society of Thoracic Surgeons Score; EuroSCORE II, European Cardiac Operative Risk Assessment System II
